# Nonsingular Stress Distribution of Edge Dislocations near Zero-Traction Boundary

**DOI:** 10.3390/ma15144929

**Published:** 2022-07-15

**Authors:** Hiroyuki Shima, Takashi Sumigawa, Yoshitaka Umeno

**Affiliations:** 1 Department of Environmental Sciences, University of Yamanashi, 4-4-37, Takeda, Kofu 400-8510, Japan; 2 Department of Energy Conversion Science, Graduate School of Energy Science, Kyoto University, Sakyo-ku, Kyoto 606-8501, Japan; sumigawa.takashi.2c@kyoto-u.ac.jp; 3 Institute of Industrial Science, The University of Tokyo, 4-6-1, Komaba, Meguro-ku, Tokyo 153-8505, Japan; umeno@iis.u-tokyo.ac.jp

**Keywords:** edge dislocation, surface effect, gradient elasticity, gauge theory, stress distribution

## Abstract

Among many types of defects present in crystalline materials, dislocations are the most influential in determining the deformation process and various physical properties of the materials. However, the mathematical description of the elastic field generated around dislocations is challenging because of various theoretical difficulties, such as physically irrelevant singularities near the dislocation-core and nontrivial modulation in the spatial distribution near the material interface. As a theoretical solution to this problem, in the present study, we develop an explicit formulation for the nonsingular stress field generated by an edge dislocation near the zero-traction surface of an elastic medium. The obtained stress field is free from nonphysical divergence near the dislocation-core, as compared to classical solutions. Because of the nonsingular property, our results allow the accurate estimation of the effect of the zero-traction surface on the near-surface stress distribution, as well as its dependence on the orientation of the Burgers vector. Finally, the degree of surface-induced modulation in the stress field is evaluated using the concept of the $L_{2}$-norm for function spaces and the comparison with the stress field in an infinitely large system without any surface.

## 1. Introduction

Continuum mechanics theory enables the calculation of the spatial distribution of stress (and strain) inside a material and its deformation under loading. If the interior of the material is ideally uniform and free from defects, then the elastic field is smooth and continuous over the system. However, in actual materials, a certain number of defects are often embedded in a discrete and inhomogeneous manner. Thus, contributions from those defects to the mechanical properties of the system in the realm of continuum theory should be considered [1,2].

Of many types of defects, dislocations are ubiquitous in crystalline metals and alloys and are typically found at a high density of $10^{8}$ or more per 1 cm^2^. Each dislocation is a line defect that disturbs the lattice arrangement of the perfect crystal, locally breaking the translational symmetry and acting as the source of stress and strain that are distributed in the materials [3]. The broken symmetry near the dislocation-core and its movement due to external forces or thermal excitations play primary roles in the physical phenomena. For instance, the plastic deformation of a crystal is caused by a large flow of dislocations [4,5], and work hardening is caused by the dislocation arrangement formed in the crystal [6]. Otherwise, the nucleation and mobility of dislocations near the tip of a crack have a strong impact on the fracture mechanics of the material [7]. Furthermore, an in-depth understanding of dislocation dynamics and dislocation-induced elastic fields near traction-free boundaries [8,9,10] and grain boundaries [9,11,12,13,14], as well as the assessment of the boundary conditions used in the existing formulations [15] are critical to predicting the performance of advanced materials: they include gold nanowires [16,17], barium titanium nanomaterials [18], functionally graded materials [19], and other metallic and semiconductor materials [20,21,22,23].

The mechanics of dislocations and their long-range correlated behaviors [24] have been addressed successfully using conventional (traditional) dislocation theory, as well as coarse-grained approaches [25], at least from a macroscopic viewpoint [26,27,28]. However, when considering nanometric-scale mechanics, conventional dislocation theory may not work adequately. The most significant drawback of the conventional theory is that stress and strain fields diverge infinitely near the dislocation-core. Because of this divergence, the theory does not fully describe the elastic field near the dislocation-core. This loss of accuracy hinders a detailed analysis of the near-core elastic field. Still, this analysis is crucial in the cases where a large number of dislocations are densely packed in a nanoscopic region or close to the interface (free surfaces, grain boundaries, etc.), as experimentally observed in the middle and late stages of the plastic deformation of metals under cyclic loading [4,29]. Furthermore, the conventional dislocation theory is intrinsically size-independent, and thus, no characteristic length appears. This complicates the theoretical interpretation of the characteristic length of the self-organized structures of dislocation ensembles that occur spontaneously during the cyclic deformation of metals [30,31].

To overcome these difficulties, various nonclassical continuum theories of dislocations have been developed in recent decades. These include theories of nonlocal elasticity [32,33], gradient elasticity [34,35], the gauge-field approach [36,37], and other sophisticated numerical approaches [38,39]. One notable feature of these nonstandard approaches is that stress singularities at the dislocation-core are eliminated in the obtained solutions so that the stress distribution becomes smooth and continuous without a divergence [33,40]. In addition, all these approaches engender characteristic length scales defined by theory-dependent parameters (e.g., nonlocality parameter, gradient coefficient, or related material parameters), which correspond to the spatial dimension of dislocation-cores. These nonsingular solutions turned out to be consistent with the numerical simulations of elastic fields realized in actual materials [41,42] and those caused by uniformly moving dislocations in an infinite body [43]. The practical significance of the nonsingular solutions has also been confirmed in the study of stress fields near crack tips in isotropic elastic materials [44]. Yet, most theoretical studies reported on the nonsingular problem so far have considered the elastic field of dislocations existing in an infinitely large system, and only a few studies have handled a finite or semi-infinite system endowed with a surface boundary. For microsized and nanosized materials [4,30], the ratio of the surface area to sample volume is so high that the effect of a surface boundary should be more prominent than that observed in macroscale samples.

Against this backdrop, we were motivated to derive a theoretical representation of the nonsingular stress field generated by an edge dislocation near the free surface of a semi-infinitely large elastic medium. The Burgers vector of the edge dislocation was assumed to be oblique to the free surface, considering that, in the actual plastic deformation process, the slip plane of edge dislocations in metals is often oriented diagonally to the load direction. Our analytical expression allows us to quantify the spatial modulation of the nonsingular stress field of an edge dislocation in the presence of a zero-traction surface boundary. The stress-field formula derived in this study provides a theoretical basis for analyzing the behavior of dislocations accumulated near the free surface and the interaction between adjacent dislocations on micrometric and nanometric scales [4,30].

## 2. Nonsingular Elastic Field in an Infinite Medium

We assume an infinitely long, straight edge dislocation that extends along the *z*-axis in the Cartesian coordinate system. The dislocation-core is located at the origin of the *x*-*y* coordinate plane. For convenience, we first consider an infinite elastic system without boundaries, and then, a semi-infinite system, including a planar free surface with no traction. Because the system spreads infinitely in the *z* direction, the edge dislocation produces a two-dimensional state of plane stress, as well as plane strain defined by $u_{z}=0$ and $du_{i}/\mathrm{dz}=0$ for i=x,y,z, where $u_{i}$ is the *i*-directional displacement of an infinitesimal element of the system.

It is known that any in-plane stress problem can be reduced to exploring an appropriate biharmonic function [45], ϕ(x,y), the so-called Airy’s stress function, that satisfies $\nabla^{4}\phi =0$. Once the solution of ϕ(x,y) in a particular domain of interest is obtained under the given boundary conditions, the stress components within the domain can be derived through partial differentiation of ϕ(x,y) as

(1) $$\sigma_{\mathrm{xx}}\left(x,y\right)=\frac{\partial^{2}\phi }{\partial y^{2}},\;\;\sigma_{\mathrm{yy}}\left(x,y\right)=\frac{\partial^{2}\phi }{\partial x^{2}},\;\;\tau_{\mathrm{xy}}\left(x,y\right)=\frac{-\partial^{2}\phi }{\partial x\partial y}.$$

The existing work based on the gradient elasticity theory [34,35] and the gauge field theory [36,37] has unveiled that Airy’s stress function ϕ(x,y) associated with a nonsingular stress field of an edge dislocation with the Burgers vector parallel to the *x*-axis obeys the following inhomogeneous Helmholtz equation:

(2) $$\left(\nabla^{2}-\kappa^{2}\right)\phi \left(x,y\right)=\kappa^{2}Gy\log\left(\sqrt{x^{2}+y^{2}}\right).$$

Here, G is a material-dependent constant defined by

(3) $$G=\frac{\mu b}{2\pi (1-\nu^{*})},$$

with the elastic shear modulus μ, the magnitude of Burgers vector *b*, and Poisson’s ratio $\nu^{*}$. The solution of Equation (2) is represented by (see Appendix A)

(4) $$\phi \left(x,y\right)=-2Gy\left[\frac{\log\left(\sqrt{x^{2}+y^{2}}\right)}{2}+\frac{1-\kappa \sqrt{x^{2}+y^{2}}\cdot K_{1}\left(\kappa \sqrt{x^{2}+y^{2}}\right)}{\kappa^{2}\left(x^{2}+y^{2}\right)}\right].$$

In Equation (4), $K_{n}$ indicates the *n*th-order modified Bessel function of the second kind (see Appendix B). By taking the partial derivative of ϕ(x,y) twice according to Equation (1), we obtain the nonsingular solution of the stress field around the edge dislocation located at the origin of an infinitely large elastic medium.

The factor κ in Equation (2), having dimension of inverse length, is decisive for the elastic field obtained from ϕ(x,y) to be free of singularities at the dislocation-core (i.e., the origin of the *x*–*y* coordinate plane). In the framework of the gauge field theory, κ is defined by the coefficient of the constitutive relation between translational gauge-invariant physical state quantities; κ determines the position and magnitude of the extrema of the nonsingular stress field. In contrast, in the gradient elasticity theory, $\kappa^{-2}$ serves as the gradient coefficient appearing in the modified constitutive relation. It should be emphasized that, in both theories, $\kappa^{-1}$ is regarded as a characteristic length scale of the system. In the limit of $\kappa^{-1}\to 0$ (or equivalently, κ→∞), the problem under consideration falls into a conventional one suffering from the dislocation-core singularity.

It is noteworthy that the nonsingular solution of ϕ(x,y) given by Equation (4) covers the classical counterpart suffered from a dislocation-core singularity, as confirmed by considering the limiting behaviors of the modified Bessel function $K_{n}\left(u\right)$. In general, $K_{n}\left(u\right)$ is a monotonic decreasing function with *u*. In particular, when n=1, its asymptotic decays for u≫1 and u≪1 are approximated by [46]

(5) $$K_{1}\left(u\right)≃\sqrt{\frac{\pi }{2u}}e^{-u}\;\left(u\gg 1\right)\;\mathrm{and}\;K_{1}\left(u\right)≃\frac{1}{u}\;\left(u\ll 1\right).$$

At $\sqrt{x^{2}+y^{2}}\gg \kappa^{-1}$, therefore, the second term in the square brackets in Equation (4) vanishes so that ϕ(x,y) reduces to the classical stress function for an edge dislocation:

$$\phi \left(x,y\right)=-Gy\log\left(\sqrt{x^{2}+y^{2}}\right).$$

## 3. Semi-Infinite Problem Solution

### 3.1. Outline of Strategy

The stress field in a semi-infinite plane with a free surface can be derived by considering the superposition of three Airy’s stress functions such that their sum satisfies the traction-free boundary condition. To proceed with this argument, let us suppose that a semi-infinitely large elastic medium spans in the region of x<0, and an edge dislocation is positioned at (x,y)=(−d,0) with d>0. If there were no free surfaces and, thus, the elastic medium was infinitely large, the stress field caused by this edge dislocation could have been derived from Airy’s stress function $\phi^{\left(\mathrm{re}\right)}\left(x,y\right)$ given by

(6) $$\phi^{\left(\mathrm{re}\right)}\left(x,y\right)=\phi \left(x+d,y\right),$$

where the superscript “re” indicates that the edge dislocation at x=−d actually exists in the real elastic material under consideration. In Equation (6), ϕ(x+d,y) is a function obtained by rewriting the argument *x* of ϕ(x,y) expressed by Equation (4) to x+d.

As a matter of course, the stress field derived from $\phi^{\left(\mathrm{re}\right)}\left(x,y\right)$ does not satisfy the traction-free boundary conditions expressed by $\sigma_{\mathrm{xx}}=0$ and $\tau_{\mathrm{xy}}=0$ along x=0. Of these two, the former requirement is accomplished by superposing another Airy’s stress function defined by

(7) $$\phi^{\left(\mathrm{im}\right)}\left(x,y\right)=\phi \left(x-d,y\right),$$

onto $\phi^{\left(\mathrm{re}\right)}\left(x,y\right)$. The superscript “im” indicates a contribution from a virtual image dislocation, which is assumed to be located at (x,y)=(+d,0) (outside the free surface), but it is not present in the real material. We also assume that the Burgers vector of the image dislocation has the same magnitude as that of the real dislocation, whereas their orientations can be different from each other. By tuning the relative orientations of the two Burgers vectors, we can make the stress field obtained from the sum of $\phi^{\left(\mathrm{re}\right)}\left(x,y\right)+\phi^{\left(\mathrm{im}\right)}\left(x,y\right)$ satisfy the condition of $\sigma_{\mathrm{xx}}\left(x,y\right)=0$ at x=0. Here, the contribution $\sigma_{\mathrm{xx}}^{\left(\mathrm{im}\right)}\left(x,y\right)$ from the image dislocation cancels out the contribution $\sigma_{\mathrm{xx}}^{\left(\mathrm{re}\right)}\left(x,y\right)$ from the real dislocation at every point along x=0. This technique of canceling the stress component $\sigma_{\mathrm{xx}}$ at a given boundary is called the image force method [47,48], which is a mechanical analog of the image charge construction employed in electromagnetism [49].

The remaining requirement of $\tau_{\mathrm{xy}}=0$ along x=0 is met by introducing a third “excess” Airy’s stress function, designated by $\phi^{\left(\mathrm{ex}\right)}\left(x,y\right)$, such that the two components of the stress field derived from it, $\sigma_{xx}^{\left(\mathrm{ex}\right)}$ and $\tau_{\mathrm{xx}}^{\left(\mathrm{ex}\right)}$, satisfy the following conditions at x=0:

(8) $$\sigma_{\mathrm{xx}}^{\left(\mathrm{ex}\right)}\left(0,y\right)=0\;\;\mathrm{and}\;\;\tau_{\mathrm{xy}}^{\left(\mathrm{ex}\right)}\left(0,y\right)=-\left\{\tau_{\mathrm{xy}}^{\left(\mathrm{re}\right)}\left(0,y\right)+\tau_{\mathrm{xy}}^{\left(\mathrm{im}\right)}\left(0,y\right)\right\}.$$

The latter in Equation (8) indicates that the contribution from the excess Airy’s function cancels out those from the two dislocations (real and image). If such $\phi^{\left(\mathrm{ex}\right)}\left(x,y\right)$ is found, the sum of three Airy’s functions given by

(9) $$\phi^{\left(\mathrm{all}\right)}\left(x,y\right)=\phi^{\left(\mathrm{re}\right)}\left(x,y\right)+\phi^{\left(\mathrm{im}\right)}\left(x,y\right)+\phi^{\left(\mathrm{ex}\right)}\left(x,y\right)$$

satisfies both $\sigma_{\mathrm{xx}}^{\left(\mathrm{all}\right)}\left(x,y\right)=0$ and $\tau_{\mathrm{xy}}^{\left(\mathrm{all}\right)}=0$ along x=0. Thus, it can be used to obtain the three stress components distributed over the semi-infinite plane at x<0.

### 3.2. Stress Due to the Real Dislocation at x=−d

In metals, edge dislocations that move in a slanted direction with respect to the free surface play a vital role in plastic deformation [4,6,30]. Therefore, we developed an explicit and tractable formula for the nonsingular stress field of a semi-infinite system to infer the surface effect on the fundamental properties of the edge dislocations near the free surface.

To simplify the mathematical expressions, we henceforth use the following notations:

(10) $$x_{p}\equiv x+d,\;c_{p}\left(x,y\right)\equiv \kappa \sqrt{{\left(x+d\right)}^{2}+y^{2}},$$

where the subscript “p” indicates the plus sign “+” contained in the term x+d. Next, we calculate the three components of the stress, $\sigma_{\mathrm{xx}}^{\left(\mathrm{re}\right)}\left(x,y\right)$, $\sigma_{\mathrm{yy}}^{\left(\mathrm{re}\right)}\left(x,y\right)$, and $\tau_{\mathrm{xy}}^{\left(\mathrm{re}\right)}\left(x,y\right)$, generated by the edge dislocation located at (x,y)=(−d,0). Herein, the Burgers vector is oriented in the slanted direction by α with respect to the *x*-axis. The coordinate rotation of the stress components is obtained by partial differentiation of $\phi^{\left(\mathrm{re}\right)}\left(x,y\right)$ as

(11) $$\sigma_{\mathrm{xx}}^{\left(\mathrm{re}\right)}\left(x,y\right)=G\frac{4\chi_{\mathrm{xx}}^{\left[1\right]}+c_{p}^{2}\chi_{\mathrm{xx}}^{\left[2\right]}+2c_{p}^{2}K_{0}\left(c_{p}\right)\chi_{\mathrm{xx}}^{\left[3\right]}+2c_{p}K_{1}\left(c_{p}\right)\chi_{\mathrm{xx}}^{\left[4\right]}}{c_{p}^{2}{\left(x_{p}^{2}+y^{2}\right)}^{2}},$$

with

(12) $$\begin{array}{ccc} \chi_{\mathrm{xx}}^{\left[1\right]} & = & x_{p}\left(x_{p}^{2}-3y^{2}\right)\sin\alpha +y\left(3x_{p}^{2}-y^{2}\right)\cos\alpha , \end{array}$$

(13) $$\begin{array}{ccc} \chi_{\mathrm{xx}}^{\left[2\right]} & = & -x_{p}\left(x_{p}^{2}-y^{2}\right)\sin\alpha -y\left(3x_{p}^{2}+y^{2}\right)\cos\alpha , \end{array}$$

(14) $$\begin{array}{ccc} \chi_{\mathrm{xx}}^{\left[3\right]} & = & -\chi_{xx}^{\left[1\right]}, \end{array}$$

(15) $$\begin{array}{ccc} \chi_{\mathrm{xx}}^{\left[4\right]} & = & -2\chi_{xx}^{\left[1\right]}+c_{p}^{2}y^{2}\left(x_{p}\sin\alpha +y\cos\alpha \right), \end{array}$$

and

(16) $$\sigma_{yy}^{\left(\mathrm{re}\right)}\left(x,y\right)=G\frac{4\chi_{yy}^{\left[1\right]}+c_{p}^{2}\chi_{yy}^{\left[2\right]}+2c_{p}^{2}K_{0}\left(c_{p}\right)\chi_{yy}^{\left[3\right]}+2c_{p}K_{1}\left(c_{p}\right)\chi_{yy}^{\left[4\right]}}{c_{p}^{2}{\left(x_{p}^{2}+y^{2}\right)}^{2}},$$

with

(17) $$\begin{array}{ccc} \chi_{yy}^{\left[1\right]} & = & -x_{p}\left(x_{p}^{2}-3y^{2}\right)\sin\alpha -y\left(3x_{p}^{2}-y^{2}\right)\cos\alpha \;\left(=\;-\chi_{xx}^{\left[1\right]}\right), \end{array}$$

(18) $$\begin{array}{ccc} \chi_{yy}^{\left[2\right]} & = & -x_{p}\left(x_{p}^{2}+3y^{2}\right)\sin\alpha +y\left(x_{p}^{2}-y^{2}\right)\cos\alpha , \end{array}$$

(19) $$\begin{array}{ccc} \chi_{yy}^{\left[3\right]} & = & -\chi_{yy}^{\left[1\right]}, \end{array}$$

(20) $$\begin{array}{ccc} \chi_{yy}^{\left[4\right]} & = & -2\chi_{yy}^{\left[1\right]}+c_{p}^{2}x_{p}^{2}\left(x_{p}\sin\alpha +y\cos\alpha \right), \end{array}$$

and

(21) $$\tau_{xy}^{\left(\mathrm{re}\right)}\left(x,y\right)=G\frac{4\chi_{\mathrm{xy}}^{\left[1\right]}+c_{p}^{2}\chi_{\mathrm{xy}}^{\left[2\right]}+2c_{p}^{2}K_{0}\left(c_{p}\right)\chi_{\mathrm{xy}}^{\left[3\right]}+2c_{p}K_{1}\left(c_{p}\right)\chi_{\mathrm{xy}}^{\left[4\right]}}{c_{p}^{2}{\left(x_{p}^{2}+y^{2}\right)}^{2}},$$

with

(22) $$\begin{array}{ccc} \chi_{\mathrm{xy}}^{\left[1\right]} & = & y\left(3x_{p}^{2}-y^{2}\right)\sin\alpha -x_{p}\left(x_{p}^{2}-3y^{2}\right)\cos\alpha , \end{array}$$

(23) $$\begin{array}{ccc} \chi_{\mathrm{xy}}^{\left[2\right]} & = & \left(x_{p}^{2}-y^{2}\right)\left(-y\sin\alpha +x_{p}\cos\alpha \right), \end{array}$$

(24) $$\begin{array}{ccc} \chi_{\mathrm{xy}}^{\left[3\right]} & = & -\chi_{xy}^{\left[1\right]}, \end{array}$$

(25) $$\begin{array}{ccc} \chi_{\mathrm{xy}}^{\left[4\right]} & = & -2\chi_{xy}^{\left[1\right]}+c_{p}^{2}\left(-x_{p}y\right)\left(x_{p}\sin\alpha +y\cos\alpha \right). \end{array}$$

Note that the three expressions in Equations (11), (16) and (21), have the same functional form, whereas the definitions of $\chi_{ij}^{\left[k\right]}$(i,j=xory,1≤k≤4) are different depending on the components. It also should be noted that, at sufficiently far distances from the dislocation-core (i.e., at ${\left(x-d\right)}^{2}+y^{2}\gg \kappa^{-2}$), only the term proportional to $\chi_{ij}^{\left[2\right]}$ remains non-negligible. Hence, the three components are reduced to the classical counterpart. These two noteworthy facts hold true for the stress components associated with the image dislocation, which will be described in the next subsection.

### 3.3. Stress Due to the Image Dislocation at x=+d

**Figure 1:**
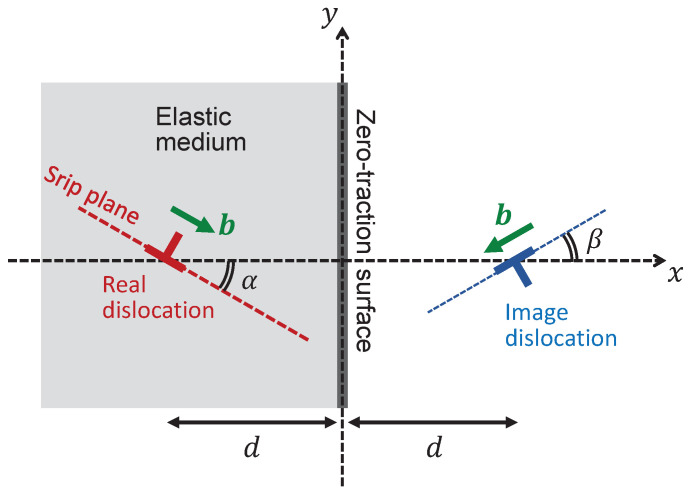
Real edge dislocation (**left**) existing inside a semi-infinite elastic medium and image edge dislocation (**right**) virtually introduced for convention. The Burgers vectors of the two dislocations are assumed to be oblique relative to the *x*-axis at angles α and β, respectively.

The three components of the stress generated by the image edge dislocation at (x,y)=(+d,0), denoted by $\sigma_{xx}^{\left(\mathrm{im}\right)}\left(x,y\right)$, $\sigma_{yy}^{\left(\mathrm{im}\right)}\left(x,y\right)$, and $\tau_{xy}^{\left(\mathrm{im}\right)}\left(x,y\right)$, can be obtained by the variable transformation for all the mathematical expressions regarding the real dislocation counterparts given in the previous subsection. Once they are rewritten as G→−G, α→β, and x+d→x−d, we obtain the expressions of the stress component of the image dislocation drawn in Figure 1.

By setting the slanted angles to α=θ and β=−θ, it is easily proven that, at x=0, the obtained expressions of $\sigma_{xx}^{\left(\mathrm{re}\right)}$ and $\sigma_{xx}^{\left(\mathrm{im}\right)}$ cancel each other out. This cancellation means that the virtually introduced image dislocation makes it possible to eliminate the $\sigma_{xx}$ component at the surface, on which no traction force must be present.

### 3.4. Sum of the Shear Stress Components at a Free Surface

For later use in the derivation of the excess Airy function $\phi^{\left(\mathrm{ex}\right)}$, we now compute the sum of the two shear components of the two above-mentioned dislocations along the line of x=0, denoted by $\tau_{xy}^{\left(\mathrm{re}\right)}\left(0,y\right)+\tau_{xy}^{\left(\mathrm{im}\right)}\left(0,y\right)$. Using the notation of

(26) $$c_{0}\left(y\right)=\kappa \sqrt{y^{2}+d^{2}},$$

the sum of the shear components is rewritten as

(27) $$\begin{array}{ccc} \tau_{xy}^{\left(\mathrm{re}\right)}\left(0,y\right)+\tau_{xy}^{\left(\mathrm{im}\right)}\left(0,y\right) & = & 2G\frac{4\omega^{\left[1\right]}+c_{0}^{2}\omega^{\left[2\right]}+2c_{0}^{2}K_{0}\left(c_{0}\right)\omega^{\left[3\right]}+2c_{0}K_{1}\left(c_{0}\right)\omega^{\left[4\right]}}{c_{0}^{2}{\left(y^{2}+d^{2}\right)}^{2}}, \end{array}$$

with

(28) $$\begin{array}{ccc} \omega^{\left[1\right]} & = & -y\left(y^{2}-3d^{2}\right)\sin\theta +d\left(3y^{2}-d^{2}\right)\cos\theta , \end{array}$$

(29) $$\begin{array}{ccc} \omega^{\left[2\right]} & = & \left(y^{2}-d^{2}\right)\left(y\sin\theta -d\cos\theta \right), \end{array}$$

(30) $$\begin{array}{ccc} \omega^{\left[3\right]} & = & -\omega^{\left[1\right]}, \end{array}$$

(31) $$\begin{array}{ccc} \omega^{\left[4\right]} & = & -2\omega^{\left[1\right]}+c_{0}^{2}\left(-dy\right)\left(d\sin\theta -y\cos\theta \right). \end{array}$$

In the limit of $c_{0}\to \infty$, the right-hand side of Equation (27) is reduced to

(32) $$2G\frac{\left(y^{2}-d^{2}\right)\left(y\sin\theta -d\cos\theta \right)}{{\left(y^{2}+d^{2}\right)}^{2}},$$

which agrees with the counterpart obtained by the classical dislocation theory.

### 3.5. Stress from Excess Airy’s Function

For now, we have successfully derived the nonsingular stress components associated with the real and image dislocations that satisfy one of the two free surface conditions, i.e., $\sigma_{xx}\left(0,y\right)=0$. In order to make another remaining component $\tau_{xy}\left(0,y\right)$ be zero keeping $\sigma_{xx}\left(0,y\right)=0$, we now consider the definition of excess Airy’s function $\phi^{\left(\mathrm{ex}\right)}\left(x,y\right)$.

The two stress components, $\sigma_{xx}^{\left(\mathrm{ex}\right)}\left(x,y\right)$ and $\tau_{xy}^{\left(\mathrm{ex}\right)}\left(x,y\right)$, associated with $\phi^{\left(\mathrm{ex}\right)}$ must satisfy the following relations:

(33) $$\sigma_{xx}^{\left(\mathrm{ex}\right)}\left(0,y\right)=0,$$

and

(34) $$\tau_{xy}^{\left(\mathrm{ex}\right)}\left(0,y\right)=\;-2G\frac{4\omega^{\left[1\right]}+c_{0}^{2}\omega^{\left[2\right]}+2c_{0}^{2}K_{0}\left(c_{0}\right)\omega^{\left[3\right]}+2c_{0}K_{1}\left(c_{0}\right)\omega^{\left[4\right]}}{c_{0}^{2}{\left(y^{2}+d^{2}\right)}^{2}}.$$

The solution satisfying the former condition can be derived by the separation of variables method, signified by $\phi^{\left(\mathrm{ex}\right)}\left(x,y\right)=X\left(x\right)Y\left(y\right)$ (see Appendix C). A straightforward calculation leads to the conclusion that the general solution of $\phi^{\left(\mathrm{ex}\right)}\left(x,y\right)$ that satisfies $\sigma_{xx}^{\left(\mathrm{ex}\right)}\left(0,y\right)=0$ is

(35) $$\phi^{\left(\mathrm{ex}\right)}\left(x,y\right)=\int_{0}^{\infty }a_{1}\left(k\right)xe^{kx}\sin ky\;dk+\int_{0}^{\infty }a_{2}\left(k\right)xe^{kx}\cos ky\;dk,$$

where $a_{1}\left(k\right)$ and $a_{2}\left(k\right)$ are *k*-dependent coefficients. Its partial differentiations according to Equation (1) yield the stress components associated with $\phi^{\left(\mathrm{ex}\right)}\left(x,y\right)$:

(36) $$\begin{array}{ccc} \sigma_{xx}^{\left(\mathrm{ex}\right)}\left(x,\;y\right) & = & I_{s,1}\left(-kx;x,y\right)+I_{c,2}\left(-kx;x,y\right), \end{array}$$

(37) $$\begin{array}{ccc} \sigma_{yy}^{\left(\mathrm{ex}\right)}\left(x,\;y\right) & = & I_{s,1}\left(2\;+\;kx;x,y\right)+I_{c,2}\left(2\;+\;kx;x,y\right), \end{array}$$

(38) $$\begin{array}{ccc} \tau_{xy}^{\left(\mathrm{ex}\right)}\left(x,\;y\right) & = & I_{s,2}\left(1\;+\;kx;x,y\right)+I_{c,1}\left(\;-\;1\;-\;kx;x,y\right), \end{array}$$

where

(39) $$\begin{array}{ccc} I_{s,i}\left(u;x,y\right) & = & \int_{0}^{\infty }u\;ka_{i}\left(k\right)\;e^{kx}\sin ky\;dk, \end{array}$$

(40) $$\begin{array}{ccc} I_{c,i}\left(u;x,y\right) & = & \int_{0}^{\infty }u\;ka_{i}\left(k\right)\;e^{kx}\cos ky\;dk, \end{array}$$

with i=1 or 2.

The remaining task is to find the *k*-dependences of $a_{1}\left(k\right)$ and $a_{2}\left(k\right)$ that suffice for $\tau_{xy}^{\left(\mathrm{ex}\right)}\left(0,y\right)$ to cancel the shear stress contribution of Equation (27) from the real and image dislocations. From Equations (34) and (38), this requirement regarding $\tau_{xy}^{\left(\mathrm{ex}\right)}\left(0,y\right)$ is expressed as

(41) $$I_{s,2}\left(1;0,y\right)+I_{c,1}\left(\;-\;1;0,y\right)\;=\;-2G\frac{4\omega^{\left[1\right]}+c_{0}^{2}\omega^{\left[2\right]}+2c_{0}^{2}K_{0}\left(c_{0}\right)\omega^{\left[3\right]}+2c_{0}K_{1}\left(c_{0}\right)\omega^{\left[4\right]}}{c_{0}^{2}{\left(y^{2}+d^{2}\right)}^{2}},$$

with the definitions of $c_{0}\left(y\right)$ and $\omega^{\left[j\right]}\left(y,\theta \right)$(j=1,2,3,4) given by Equations (26) and (29)–(31), respectively. We then apply the inverse cosine and sine Fourier transforms to both sides of Equation (41), in which the integrations are performed using Cauchy’s residue theorem, which is a powerful tool for evaluating real improper integrals (from *−∞* to *∞*) of analytic functions. As a result of the calculation, we obtain the following solution:

(42) $$\begin{array}{ccc} ka_{1}\left(k\right)\; & = & \;2G\left(kd-\frac{2k^{2}}{\kappa^{2}}\right)e^{-kd}\cos\theta +\frac{4G}{\pi }\;\int_{-\infty }^{\infty }\;K\left(y,\theta \right)\cos ky\;dy, \end{array}$$

(43) $$\begin{array}{ccc} ka_{2}\left(k\right)\; & = & \;2G\left(kd-1-\frac{2k^{2}}{\kappa^{2}}\right)e^{-kd}\sin\theta -\frac{4G}{\pi }\;\int_{-\infty }^{\infty }\;K\left(y,\theta \right)\sin ky\;dy. \end{array}$$

with

(44) $$K\left(y,\theta \right)=\frac{c_{0}^{2}K_{0}\left(c_{0}\right)\omega^{\left[3\right]}+c_{0}K_{1}\left(c_{0}\right)\omega^{\left[4\right]}}{c_{0}^{2}{\left(y^{2}+d^{2}\right)}^{2}},\;c_{0}=\kappa \sqrt{y^{2}+d^{2}}.$$

The integrals in Equations (42) and (43) cannot be solved analytically owing to the presence of $K_{0}$ and $K_{1}$. Therefore, we used numerical integration to derive the *k*-dependences of the coefficients $ka_{1}\left(k\right)$ and $ka_{2}\left(k\right)$. Once $ka_{1}\left(k\right)$ and $ka_{2}\left(k\right)$ are numerically obtained, the spatial distribution of the stress components, $\sigma_{xx}^{\left(\mathrm{ex}\right)}\left(x,\;y\right)$, $\sigma_{yy}^{\left(\mathrm{ex}\right)}\left(x,\;y\right)$, and $\tau_{xy}^{\left(\mathrm{ex}\right)}\left(x,\;y\right)$, can be computed using Equations (36)–(40). Eventually, we obtain the nonsingular stress field of the edge dislocation in the infinitely large system with a traction-free planar surface.

## 4. Numerical Results

### 4.1. Nonsingular Stress Field of an Edge Dislocation

**Figure 2:**
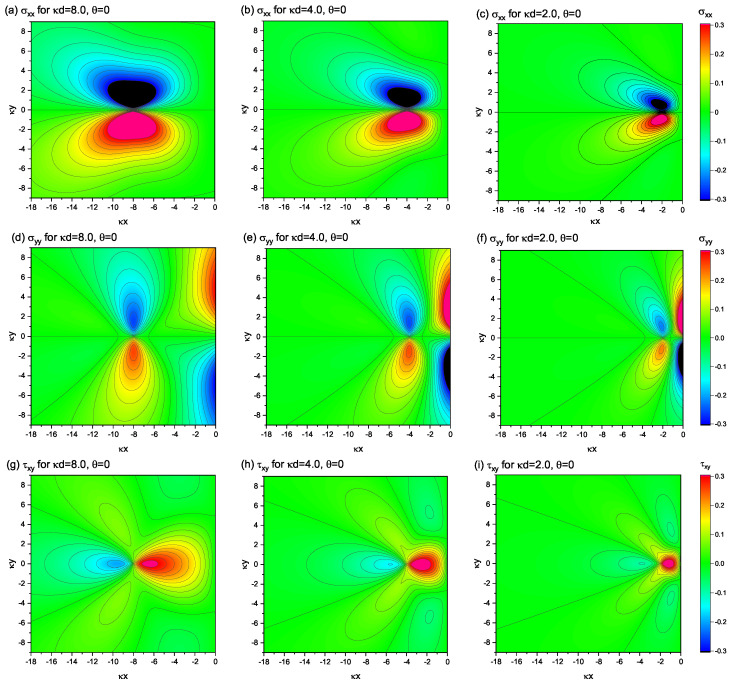
Nonsingular stress fields around a single edge dislocation positioned at (x,y)=(−d,0) with various values of *d*. For all the plots, θ=0 is fixed, indicating that the Burgers vector of the dislocation is assumed to be parallel to the *x*-axis. Upper row: (**a**–**c**) $\sigma_{xx}\left(x,y\right)$. Middle row: (**d**–**f**) $\sigma_{yy}\left(x,y\right)$. Bottom row: (**g**–**i**) $\tau_{xy}\left(x,y\right)$. The units of stress and length scales are set to be $\mu \kappa b/2(1-\nu^{*})$ and $\kappa^{-1}$, respectively.

Figure 2 shows the contour plots of the nonsingular stress distribution in a semi-infinite elastic medium depicted in Figure 1. The panels (a)–(c) show the component of $\sigma_{xx}$; (d)–(f) show $\sigma_{yy}$; (g)–(i) show $\tau_{xy}$. The Burgers vector orientation is fixed to be parallel to the *x*-axis. The distances from the free surface to the dislocation-core measured by κd (i.e., the ratio of *d* to the characteristic length scale $\kappa^{-1}$) are shifted as κd=8.0, 4.0, and 2.0, from left to right in the figure.

When κd=8.0, the profiles of the stress distribution are fairly close to those realized in an infinitely large system with no boundary, in particular for the components of $\sigma_{xx}$ and $\tau_{xy}$. The profile of $\sigma_{xx}$ in Figure 2a is close to a figure-eight-shaped one, endowed with the inversion symmetry with respect to the *x*-axis. The profile of $\tau_{xy}\left(x,y\right)$ in Figure 2g also exhibits a multipolar symmetry, analogous to the infinite system. An exception is the profile of $\sigma_{xy}$, where strong amplitude stresses occur near the free surface (all but y=0), even though the dislocation is far from the surface.

**Figure 3:**
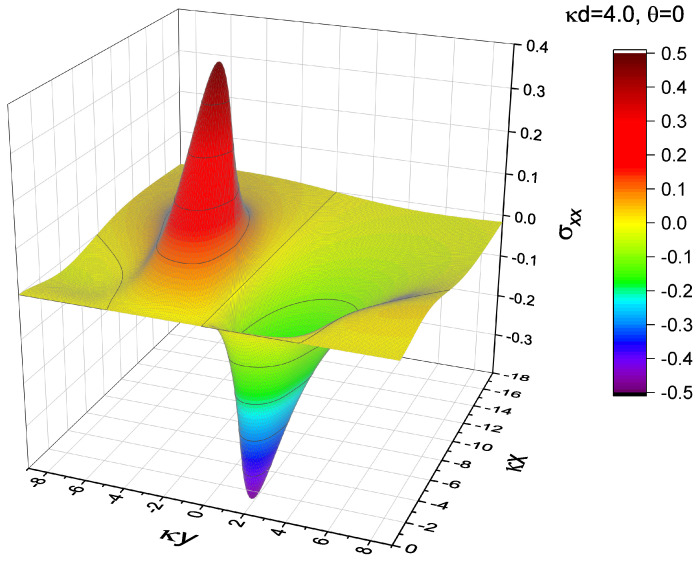
Three-dimensional plot of $\sigma_{xx}$ for the case of κd=4.0 and θ=0, which is shown in Figure 2b. The unit of stress is $\mu \kappa b/2(1-\nu^{*})$. The smoothness and continuity in the stress field with no singularity near the dislocation-core can be visually confirmed.

As the dislocation approaches the surface, the stress field distribution gradually deviates from that in the infinite system. The area with a high stress magnitude gradually decreases with decreasing κd, indicating that the presence of the free surface suppresses the near-surface field. However, regardless of the proximity of the dislocation to the surface, the divergence near the dislocation-core that occurs in the classical theory does not occur here. The stress field derived in this work is always smooth and continuous, differing from the stress field derived from the classical theory, wherein the upward and downward peak magnitudes diverge infinitely, resulting in the field discontinuity at y=0. The disappearance of the singularity in our results can be visually observed in the 3D plot presented in Figure 3, where the component $\sigma_{xx}$ under the settings of κd=4.0 and θ=0 (the same as in Figure 2b) is plotted as an example.

**Figure 4:**
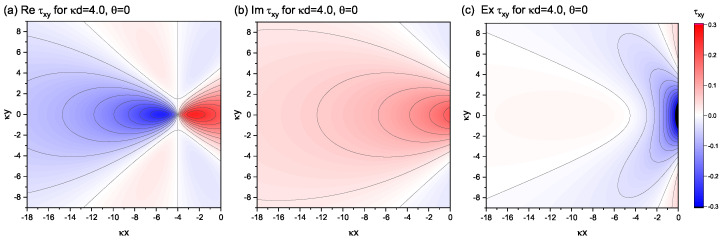
Contour diagram decomposing the contributions from (**a**) $\tau_{xy}^{\left(\mathrm{re}\right)}$, (**b**) $\tau_{xy}^{\left(\mathrm{im}\right)}$, and (**c**) $\tau_{xy}^{\left(\mathrm{ex}\right)}$ to $\tau_{xy}^{\left(\mathrm{all}\right)}$ for the case of κd=4.0 and θ=0, which is shown in Figure 2h. The unit of stress is $\mu \kappa b/2(1-\nu^{*})$.

Figure 4 depicts contour maps, which decompose the three constituent elements of the shear component, $\tau_{xy}^{\left(\mathrm{re}\right)}$, $\tau_{xy}^{\left(\mathrm{im}\right)}$, and $\tau_{xy}^{\left(\mathrm{ex}\right)}$, under the settings of κd=4.0 and θ=0. The superposition of the three contour plots reproduces Figure 2h. The stress distribution drawn by $\tau_{xy}^{\left(\mathrm{re}\right)}$ would have been realized by the edge dislocation at x=−d if there was no surface boundary at x=0; thus, the system was infinitely large. Similarly, the contour diagram shown by $\tau_{xy}^{\left(\mathrm{im}\right)}$ is the stress field created by the (image) dislocation at x=d if the surface boundary does not exist. The third element, $\tau_{xy}^{\left(\mathrm{ex}\right)}$, is a compensating element so that the sum of the shear stress contributions from the first two elements becomes zero at the surface boundary. In fact, for the case shown in Figure 4, the large negative shear stress of $\tau_{xy}^{\left(\mathrm{ex}\right)}$ within the region near the origin compensates for the large positive shear stress derived from the sum of the other two elements, $\tau_{xy}^{\left(\mathrm{re}\right)}+\tau_{xy}^{\left(\mathrm{im}\right)}$ in the same region. It should be emphasized that all three elements are smooth and continuous, free from singularities near the dislocation-core.

**Figure 5:**
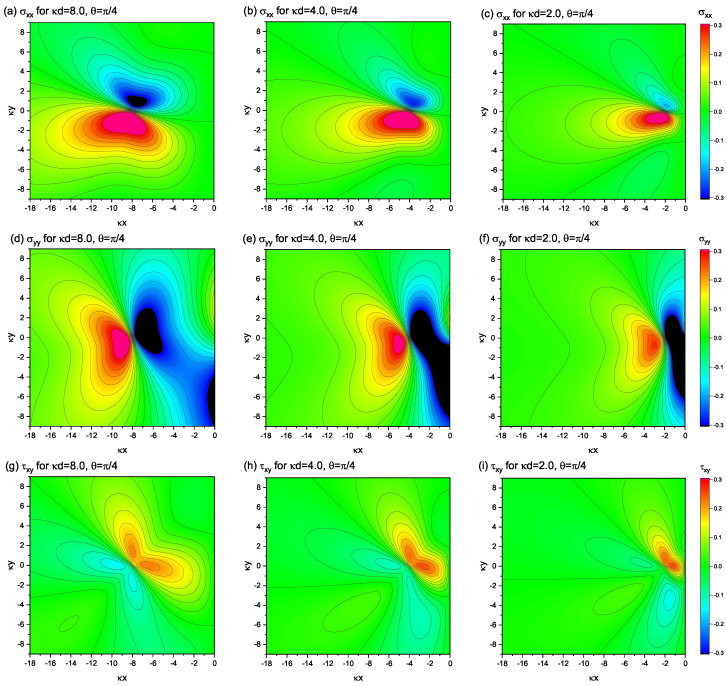
Nonsingular stress fields of an edge dislocation at (x,y)=(−d,0) with the angle θ=π/4 between the Burgers vector and the *x*-axis. Upper row: (**a**–**c**) $\sigma_{xx}\left(x,y\right)$. Middle row: (**d**–**f**) $\sigma_{yy}\left(x,y\right)$. Bottom row: (**g**–**i**) $\tau_{xy}\left(x,y\right)$. The units of stress and length scales are the same as in Figure 2.

The above-mentioned facts are valid even if we set the Burgers vector to a different orientation from the *x*-axis. Figure 5 shows the case of θ=π/4, in which the Burgers vector is oblique diagonally to the free surface. Again, the obtained stress fields are free from singularities, for any choice of κd and θ.

**Figure 6:**
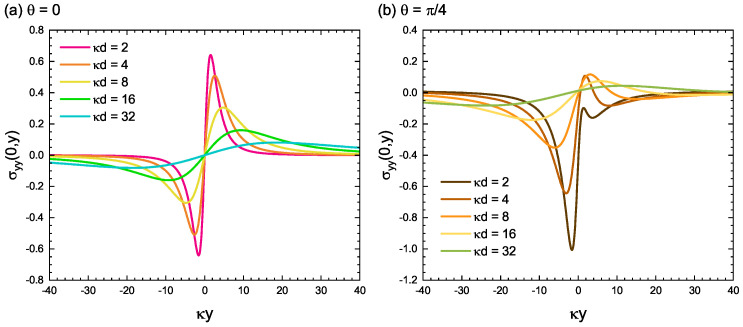
Spatial distribution of the stress component $\sigma_{yy}\left(0,y\right)$ along the free surface of x=0. The closer the dislocation is to the surface, the sharper are the peaks near y=0. The relative angle of the Burgers vector to the normal of the free surface is set to (**a**) θ=0 and (**b**) θ=π/4.

As previously mentioned, only component $\sigma_{yy}$ can have a finite magnitude at the traction-free surface at x=0. Figure 6 shows the spatial distribution of the stress component $\sigma_{yy}\left(0,y\right)$ along the free surface of x=0. The closer the dislocation is to the surface, the sharper are the peaks near y=0. As is clear from Figure 6, the regions with strong $\sigma_{yy}$ components do not disappear and remain, even if the dislocations are far away from the free surface. This result can be said to be a manifestation of the long-range interaction nature between dislocations and free surfaces.

### 4.2. Spatial Field Modulation Induced by the Free Surface

In order to estimate the magnitude of spatial modulation in the stress field caused by the presence of the zero-traction surface, we use the concept of the $L_{2}$-norm of the function [50]. In general, the $L_{2}$-norm of a function f(x,y), denoted by ${∥f∥}_{2}$, is defined by

(45) $${∥f∥}_{2}\equiv {\left\{\int_{-\infty }^{\infty }dx\int_{-\infty }^{\infty }dy{\left|f\left(x,y\right)\right|}^{2}\right\}}^{\frac{1}{2}}.$$

This quantity can be regarded as a generalization of the length of a vector $x=(x_{1},x_{2},\ldots ,x_{n})$, in an *n*-dimensional space:

(46) $$\left|x\right|={\left(\sum_{j=1}^{n}x_{j}^{2}\right)}^{\frac{1}{2}}.$$

Using this analogy, the *distance* between two functions f(x,y) and g(x,y) is measured by the $L_{2}$-norm of ${∥f-g∥}_{2}$, which quantifies the deviation of the spatial distribution of f(x,y) from that of g(x,y). Based on the discussion, we calculated the following three quantities:

(47) $$S_{\mathrm{xx}}=\frac{∥\sigma_{\mathrm{xx}}-\sigma_{\mathrm{xx}}^{\left(\mathrm{re}\right)}{∥}_{2}}{∥\sigma_{\mathrm{xx}}^{\left(\mathrm{re}\right)}{∥}_{2}},\;S_{\mathrm{yy}}=\frac{∥\sigma_{\mathrm{xy}}-\sigma_{\mathrm{xy}}^{\left(\mathrm{re}\right)}{∥}_{2}}{∥\sigma_{\mathrm{xy}}^{\left(\mathrm{re}\right)}{∥}_{2}},\;T_{xy}=\frac{∥\tau_{\mathrm{xy}}-\tau_{\mathrm{xy}}^{\left(\mathrm{re}\right)}{∥}_{2}}{∥\tau_{\mathrm{xy}}^{\left(\mathrm{re}\right)}{∥}_{2}},$$

in all of which the denominator takes the role of “unit length” for normalization.

**Figure 7:**
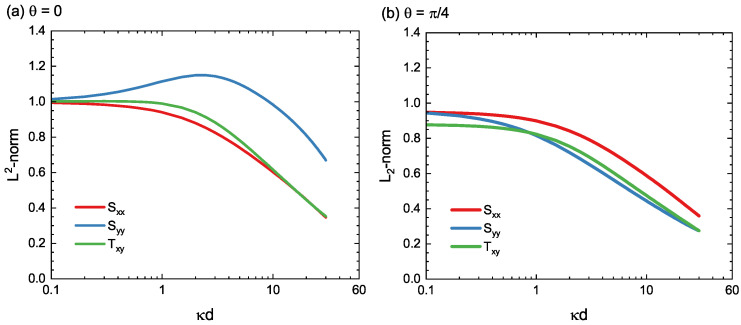
$L_{2}$-norm of the stress distribution from those produced by the real edge dislocation; see Equation (47) for the definitions of the $L_{2}$-norm. (**a**) θ=0, (**b**) θ=π/4.

Figure 7 shows the results of $S_{\mathrm{xx}}$, $S_{\mathrm{yy}}$, and $T_{\mathrm{xy}}$ for the cases of θ=0 and π/4. As can be seen from the figure, the $L_{2}$-norm curves exhibit crossover behavior across κd=1. When the dislocation is close to the surface ($d\ll \kappa^{-1}$), the $L_{2}$-norms are nearly constant. This indicates that the norm value does not change significantly because the surface effect is already sufficiently strong even if the distance from the dislocation to the surface changes slightly. In contrast, when the dislocation is far from the surface ($d\gg \kappa^{-1}$), the norm decreases in a logarithmic manner. This logarithmic heavy-tail decay in the norm represents again the long-range nature of the dislocation-to-surface interaction.

## 5. Discussion

In general, the modified Bessel functions of the second order, $K_{n}\left(u\right)$, are difficult to manipulate in an analytic manner because they cannot be expressed in a closed form. However, if the value of the variable *u* is not less than 1, the following approximation formula holds for the cases of n=0 and n=1:

(48) $$\begin{array}{ccc} K_{0}\left(u\right) & ≃ & \sqrt{\frac{\pi }{2u}}e^{-u}\left(1-\frac{1}{8u}\right). \end{array}$$

(49) $$\begin{array}{ccc} uK_{1}\left(u\right) & ≃ & \sqrt{\frac{\pi u}{2}}e^{-u}\left(1+\frac{3}{8u}\right). \end{array}$$

Therefore, when considering the case where the dislocation-core is located farther than the characteristic length $\kappa^{-1}$ from the free surface at x=0, the functions $K_{0}$ and $K_{1}$ involved in the present study can be approximated by Equations (48) and (49), respectively. This approximation will facilitate the analytical treatment of Airy’s stress functions and the resulting stress fields in the systems under study.

**Figure 8:**
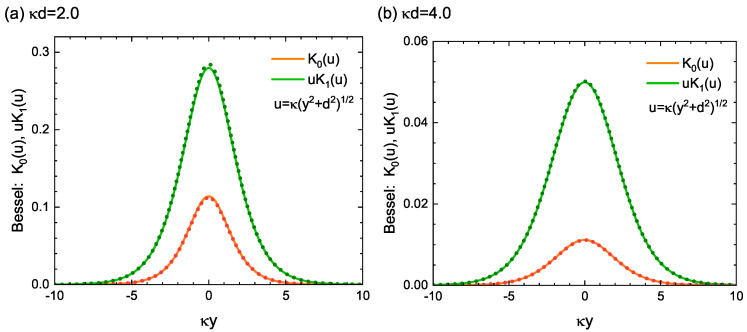
Symmetric peak of $K_{0}\left(u\right)$ and $uK_{1}\left(u\right)$ around the point of y=0 (solid curve). The variable *u* is defined by $u=\kappa \sqrt{y^{2}+d^{2}}$. Approximated curves, given by Equations (48) and (49), are also plotted by dotted curves. The edge dislocation is assumed to be located at (x,y)=(−d,0) with (**a**) d=2.0 and (**b**) d=4.0.

Figure 8 visually shows the high accuracy of this approximate expression. It shows the *y*-dependences of the functions:

(50) $$K_{0}\left(\kappa \sqrt{y^{2}+d^{2}}\right)\;\;\mathrm{and}\;\;\kappa \sqrt{y^{2}+d^{2}}K_{1}\left(\kappa \sqrt{y^{2}+d^{2}}\right),$$

both of which play an important role in the formulation of the present nonsingular stress field. Clearly, in the settings of κd=2.0 and 4.0, these functions agree quite well with the approximation for all *y* values, as drawn by dotted curves. The accuracy of the approximation increases as the value of κd increases. In addition, these functions exhibit large values only in a limited area near the origin (i.e., y=0), showing a single peak at y=0 and decaying sharply as they move away from y=0. Such a localization property, as well as the high accuracy of the approximation formula of the modified Bessel functions for κd>1 are useful in situations where simple mathematical expressions can facilitate understanding of the process under study.

## 6. Concluding Remark

We developed an explicit formula of the nonsingular stress distribution around an edge dislocation near the traction-free surface. Our formula is applicable to any value of the distance from the free surface to the dislocation center and for any orientation of the Burgers vector of the edge dislocation. The formulas allow us to evaluate the impact of the free surface on the interior stress field with high accuracy without the singularity problem that occurs at the dislocation-core in existing theories.

In this study, we only considered the stress field of single edge dislocations that exist alone, with a particular emphasis on the explicit and tractable mathematical expressions. We believe that an extension of our formulation from the single-dislocation system to mutually interacting dislocations systems will provide a theoretical basis for accomplishing singularity-free analysis of energetically stable configurations of multiple dislocations near the surface boundary of nanometals. Another possible extension of this work is related to the selection of boundary conditions. The main assumption used in this study was that the boundary of the elastic medium was a free surface with a planar geometry. The zero-traction conditions were expressed by $\sigma_{xx}=0$ and $\tau_{xy}=0$ over the boundary surface of x=0. On the other hand, actual metal specimens are often surface-treated; to deal with the latter case, appropriate boundary conditions different from those above need to be applied in theoretical analyses. Given the generality of the formulation used in this study, it can be extended to apply to the latter systems by replacing the imposed boundary conditions with appropriate ones; this issue will be considered in the near future.

## Data Availability

The data presented in this study are available upon request from the corresponding author.

## Appendix A. Solution Method of Inhomogeneous Helmholtz Equation

The inhomogeneous Helmholtz Equation (2) is solved by substituting it with the ansatz solution of [36]:

(A1) $$\phi \left(x,y\right)=-Gy\log\sqrt{x^{2}+y^{2}}+f_{1}\left(x,y\right).$$

We then have

(A2) $$\left(\nabla^{2}-\kappa^{2}\right)f_{1}\left(x,y\right)=2G\frac{\partial }{\partial y}\log\sqrt{x^{2}+y^{2}}.$$

To evaluate $f_{1}\left(x,y\right)$, we introduce $f_{2}\left(x,y\right)$ using the relation

(A3) $$f_{1}\left(x,y\right)=2G\frac{\partial }{\partial y}f_{2}\left(x,y\right).$$

From Equations (A2) and (A3), we have

(A4) $$\left(\nabla^{2}-\kappa^{2}\right)f_{2}\left(x,y\right)=\log\sqrt{x^{2}+y^{2}},$$

or equivalently, in a polar coordinate expression as

(A5) $$\left(\nabla^{2}-\kappa^{2}\right)f_{2}\left(r,\theta \right)=\log r.$$

We now remind that $K_{0}\left(u\right)$ is a solution of the following differential equation:

(A6) $$\frac{d^{2}f\left(u\right)}{du^{2}}+\frac{1}{u}\frac{df(u)}{du}-f\left(u\right)=0.$$

Comparing the latter two equations, we obtain

(A7) $$f_{2}\left(x,y\right)=-\frac{1}{\kappa^{2}}\left\{\log\sqrt{x^{2}+y^{2}}+K_{0}\left(\kappa \sqrt{x^{2}+y^{2}}\right)\right\}$$

Finally, by summarizing the results of Equations (A1), (A3) and (A7), we obtain the solution ϕ(x,y) of the original inhomogeneous Helmholtz equation, as shown in Equation (4).

## Appendix B. Properties of the Modified Bessel Function $K_{n}\left(u\right)$

The *n*-th-order modified Bessel function of the second kind, denoted by $K_{n}\left(x\right)$, is defined by one of the solutions to the modified Bessel differential equation:

(A8) $$u^{2}\frac{d^{2}f}{du^{2}}+u\frac{df}{du}-\left(u^{2}+n^{2}\right)f=0.$$

The function $K_{n}\left(u\right)$ is an important special function used in mathematics and physics; in the last few years, it has also been actively used in the fields of biometrics [51,52] and machine learning [53], in particular in association with the normal inverse Gaussian-type probability distribution [54].

The sum rule for $K_{n}\left(u\right)$ is

(A9) $$\begin{array}{ccc} K_{n}\left(u\right) & = & {\left(-1\right)}^{n+1}I_{n}\left(u\right)\log\frac{u}{2}\\ & & +\frac{{\left(-1\right)}^{n}}{2}\sum_{p=0}^{\infty }\frac{1}{p!(n+p)!}{\left(\frac{u}{2}\right)}^{2p+n}\left[\psi (p+1)+\psi (n+p+1)\right]\\ & & +\frac{1-\delta_{n,0}}{2}\sum_{p=0}^{n-1}{\left(-1\right)}^{p}\frac{(n-p-1)!}{p!}{\left(\frac{u}{2}\right)}^{2p-n}, \end{array}$$

where the function $I_{n}\left(x\right)$ appearing in the first term on the right side is called the *n*-th-order modified Bessel function of the first order, which is defined by

(A10) $$I_{n}\left(u\right)={\left(\frac{u}{2}\right)}^{n}\sum_{p=0}^{\infty }\frac{1}{p!(n+p)!}{\left(\frac{u}{2}\right)}^{2p}.$$

In Equation (A9), $\delta_{i,j}$ is Kronecker’s delta and ψ(n) is the digamma function defined by

(A11) $$\psi \left(n\right)=-\gamma +\left(1-\delta_{n,1}\right)\sum_{j=1}^{n-1}\frac{1}{j}\;\left(n\geq 1\right),$$

with γ being Euler’s constant:

(A12) $$\gamma =\lim_{n\to \infty }\left(\sum_{j=1}^{n}\frac{1}{j}-\log n\right)≃0.57721.$$

The derivative of $K_{n}\left(u\right)$ with respect to *u* follows the recurrence formula:

(A13) $$\frac{dK_{n}\left(u\right)}{du}\;\;=-\frac{n}{u}K_{n}\left(u\right)-K_{n-1}\left(u\right)\;\;=\frac{n}{u}K_{n}\left(u\right)-K_{n+1}\left(u\right).$$

More generally, it is known that

(A14) $${\left(\frac{1}{u}\frac{d}{du}\right)}^{k}\left[x^{n}K_{n}\left(u\right)\right]={\left(-1\right)}^{k}x^{n-k}K_{n-k}\left(u\right).$$

The formulae imply the following relation between adjacent trinomials:

(A15) $$K_{n+1}\left(u\right)-\frac{2n}{u}K_{n}\left(u\right)-K_{n-1}\left(u\right)=0.$$

## Appendix C. Method of the Variable Separation

In Appendix C, we derive a solution for $\phi^{\left(\mathrm{ex}\right)}\left(x,y\right)$, given by Equation (35). This solution satisfies both the conditions of Equations (33) and (34). First, we hypothesize that such a solution can be obtained using variable separation:

(A16) $$\phi^{\left(\mathrm{ex}\right)}\left(x,y\right)=X\left(x\right)Y\left(y\right).$$

Because any Airy’s stress function obeys the biharmonic relation of $\nabla^{4}\phi^{\left(\mathrm{ex}\right)}\left(x,y\right)=0$, the two functions X(x) and Y(y) must satisfy

(A17) $$\frac{\partial^{4}X}{\partial x^{4}}+\frac{2}{Y}\;\frac{\partial^{2}X}{\partial x^{2}}\;\frac{\partial^{2}Y}{\partial y^{2}}+\frac{X}{Y}\;\frac{\partial^{4}Y}{\partial y^{4}}=0,$$

which implies that the two terms:

(A18) $$\frac{2}{Y}\;\frac{\partial^{2}Y}{\partial y^{2}}\;\mathrm{and}\;\frac{1}{Y}\;\frac{\partial^{4}Y}{\partial y^{4}}$$

are constants (i.e., independent of *y*). If the first term in Equation (A18) is set to be equal to constant $-2k^{2}$ with k>0, Y(y) becomes

(A19) $$Y\left(y\right)=a_{1}^{*}\sin ky+a_{2}^{*}\cos ky,$$

with appropriate constants $a_{1}^{*}$ and $a_{2}^{*}$. Substituting Equation (A19) into (A17), we have

(A20) $$\frac{\partial^{4}X}{\partial x^{4}}-2k^{2}\frac{\partial^{2}X}{\partial x^{2}}+k^{4}X=0,$$

the solutions of which are

(A21) $$X\left(x\right)=\left(a_{3}^{*}+a_{4}^{*}x\right)e^{kx}+\left(a_{5}^{*}+a_{6}^{*}x\right)e^{-kx}\;\left(k>0\right).$$

As the stress field produced within the elastic medium far from the free surface should converge to zero, X(x) must vanish at the limit of x→−∞, which implies that $a_{5}^{*}=a_{6}^{*}=0$. In addition, the traction-free condition at the surface, $\sigma_{xx}^{\left(\mathrm{ex}\right)}\left(0,y\right)=0$, is satisfied only if

(A22) $$X\left(x\right)\frac{\partial^{2}Y\left(y\right)}{\partial y^{2}}=0$$

at x=0. Therefore, X(x) should vanish at x=0, implying that $a_{3}^{*}=0$. As a consequence, only the term attached to $a_{4}^{*}$ in Equation (A21) remains, and we obtain the solution of $\phi^{\left(\mathrm{ex}\right)}\left(x,y\right)$ as given by Equation (35).
